# Ketamine Does Not Exert Protective Properties on Dopaminergic Neurons in the Lactacystin Mouse Model of Parkinson’s Disease

**DOI:** 10.3389/fnbeh.2018.00219

**Published:** 2018-09-19

**Authors:** Lauren Deneyer, Ann Massie, Eduard Bentea

**Affiliations:** Center for Neurosciences (C4N), Department of Pharmaceutical Biotechnology and Molecular Biology, Vrije Universiteit Brussel, Brussels, Belgium

**Keywords:** isoflurane, ketamine, lactacystin, dopamine, Parkinson’s disease

## Abstract

Parkinson’s disease (PD) is an age-related neurodegenerative condition characterized by a progressive loss of dopaminergic neurons in the substantia nigra pars compacta (SNpc). A loss of proteasome function participates to the pathogenesis of PD, leading to the development of rodent models in which a proteasome inhibitor is applied to the nigrostriatal pathway. We recently characterized the intranigral lactacystin (LAC) mouse model, leading to nigrostriatal degeneration, motor dysfunction and alpha-synuclein accumulation. In the present study, we compared the effect of two commonly used anesthetics for generating animal models of PD—i.e., ketamine (KET) and isoflurane (ISO)—on the vulnerability of mouse dopaminergic neurons to proteasome inhibition-induced degeneration. Both anesthetics have the potential to affect the susceptibility of the nigrostriatal pathway for toxin-induced degeneration, and are known to modulate dopamine (DA) homeostasis. Yet, their impact on nigrostriatal degeneration in the proteasome inhibition model has not been evaluated. Unilateral injection with LAC in the SNpc of mice induced motor impairment and significantly reduced the number of dopaminergic cells to ~55%, irrespective of the anesthetic used. However, LAC-induced striatal DA depletion was slightly affected by the choice of anesthetic, resulting in a significant increase in DA turnover in the ISO- but not in KET-treated mice. These results suggest that the extent of nigrostriatal dopaminergic neural loss caused by LAC is not influenced by the choice of anesthetic, and that compared to other PD models, KET is not neuroprotective in the LAC model.

## Introduction

The main pathological hallmark of Parkinson’s disease (PD), an age-related chronic and progressive neurodegenerative disorder, is the loss of dopaminergic cells in the substantia nigra pars compacta (SNpc) and the reduction in striatal dopamine (DA) content. Proteasomal dysfunction, leading to aberrant protein turnover and build-up of misfolded or damaged proteins, has emerged as a potential contributor to cell death in PD (Poewe et al., 2017) and might be linked to the accumulation of both non-ubiquitinated and ubiquitinated proteins in the SNpc and in Lewy bodies of PD patients (McNaught et al., 2001). Accordingly, administration of lactacystin (LAC), a selective proteasome inhibitor, leads to dopaminergic cell death when applied to the nigrostriatal pathway of rodents (Mackey et al., 2013; Savolainen et al., 2017). We recently reported that intranigral administration of LAC—reflecting PD pathology where proteasome dysfunction is limited to the SN (McNaught et al., 2003)—leads to acute and non-progressive dopaminergic cell loss in mice (Bentea et al., 2015). Intracerebral injections necessitate the use of anesthetics that are known to modulate DA homeostasis including release and metabolism (Nishimura and Sato, 1999; Adachi et al., 2005; Kokkinou et al., 2018), and to potentially be either neuroprotective or neurotoxic (Peltoniemi et al., 2016). The present study aimed at comparing the susceptibility of the nigrostriatal pathway for proteasome inhibition-induced degeneration in mice that were anesthetized using either the commonly used injectable anesthetic ketamine (KET) or the volatile anesthetic isoflurane (ISO). KET is an antalgic anesthetic and non-competitive antagonist of the NMDA receptor. ISO, like most inhaled anesthetics, enhances GABA_A_ receptor function and prolongs the inhibitory postsynaptic potential. In addition to the effects on GABA_A_ receptors, the volatile anesthetics depress excitatory synaptic transmission presynaptically, where their principal action appears to be a reduction in glutamate release (Hemmings et al., 2005).

## Materials and Methods

### Animals

C57BL/6J male mice (Charles River Laboratories, France), 28–29 weeks of age at lesion, were group-housed in a 14/10 h light/dark cycle, with free access to food and tap water. Temperature (21–25°C) and relative humidity (30%–60%) were maintained constant during the experiments, which were carried out according to the Belgian animal welfare legislation (Royal Decree of 29 May 2013) and the regulations covering animal experimentation in the EU (European Communities Council Directive 2010/63/EU). The experiments were carried out in accordance to the national guidelines on animal experimentation and approved by the Ethical Committee for Animal Experiments of the Vrije Universiteit Brussel.

### Anesthetics

Mice were divided into two treatment groups, receiving either an i.p. injection of a mixture of KET (100 mg/kg; KET 1000 Ceva, Ceva Sante Animale, Belgium) and xylazine (10 mg/kg; Rompun 2%, Bayer N.V., Brussels, Belgium) or 5% ISO (Iso-vet^®^, 1,000 mg/g ISO, Dechra Veterinary Products, Netherlands) for 2 min in an induction chamber, after which anesthesia was maintained during the entire duration of the surgery (±1 h per animal) at 2.5%–3% ISO.

### Stereotaxic Surgery

Three microgram LAC (or vehicle for the sham control group) was stereotactically injected in the left substantia nigra (Bentea et al., 2015) under ISO or KET anesthesia, leading to four experimental groups: ISO LAC, ISO SHAM, KET LAC, KET SHAM (*n* = 8/group). The incidence of post-operative mortality was 1/8 for KET SHAM (12.5%), 2/8 for ISO SHAM (25%), 3/8 for KET LAC (37.5%) and 0/8 for ISO LAC (0%), resulting in a group size of *n* = 7 KET SHAM, *n* = 6 ISO SHAM, *n* = 5 KET LAC and *n* = 8 ISO LAC.

### Assessment of Motor Function

Motor function was evaluated in an accelerated rotarod test (TSE RotaRod Advanced, TSE systems) as described before (Bentea et al., 2015). Prior to surgery, mice were trained on the rotarod for 2 days. Seven days after surgery, mice were tested again to evaluate motor impairment.

### Neurochemical Analysis of Total Dopamine Content in the Striatum

Mice were sacrificed by cervical dislocation and brains were quickly removed. From the rostral part of the brain, striata were collected, weighed and homogenized in 400 μL antioxidant solution (0.05 M HCl, 0.5% Na_2_S_2_O_5_, 0.05% Na_2_ EDTA), containing 10 ng/100 μL 3,4-dihydroxybenzylamine as internal standard. Samples were analyzed for DA, 3,4-dihydroxyphenylacetic acid (DOPAC) and homovanillic acid (HVA) as described before (Massie et al., 2011).

### Quantification of Dopaminergic Cells by Immunohistochemistry

The caudal part of the brain was post-fixed for 3 days in freshly prepared 4% paraformaldehyde (Sigma-Aldrich, Brussels, Belgium), sliced in 40 μm sections using a vibratome and serially stored in 0.1 M PBS supplemented with 0.01% sodium azide at 4°C. Six slices per brain (Fu et al., 2012), covering the SNpc (−2.92 mm to −3.60 mm relative to Bregma), were selected for staining, as described before (Bentea et al., 2015; Massie et al., 2011). The number of tyrosine hydroxylase (TH)^+^ profiles was determined in the selected sections using ImageJ software (U.S. National Institutes of Health, Bethesda, MD, USA).

### Statistical Analysis

Data were expressed as mean ± standard error of the mean (SEM). Statistical analysis was performed using GraphPad Prism 6.01 software. For analysis of multiple variables within multiple groups of animals we applied two-way ANOVA followed by Tukey’s *post hoc* test. The α-value was set at 0.05.

## Results

### Influence of Anesthetics on Nigrostriatal Degeneration

**Figure 1:**
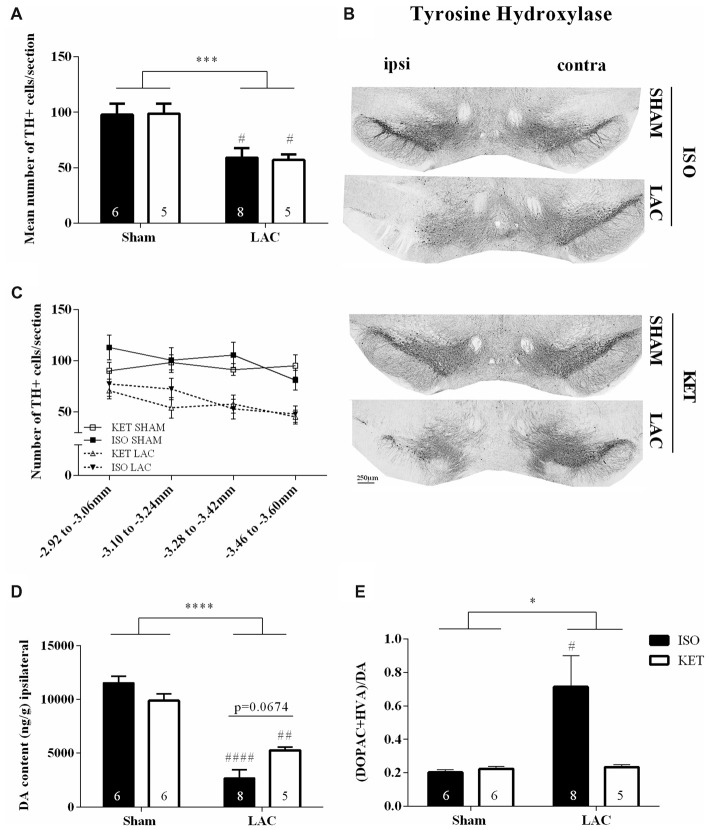
Influence of anesthetics on nigrostriatal degeneration. Ipsilateral effects of LAC-infusion on the mean number of dopaminergic cells (TH+ cells) per section in the Substantia nigra pars compacta (SNpc; **A**), striatal DA content **(D)** and DA turnover **(E)** of mice anesthetized with ISO or KET during stereotaxic surgery. For all parameters, a significant global effect of LAC lesion was present in both anesthetized groups compared to sham-lesion. At the level of the striatum, DA depletion was more pronounced in mice anesthetized with ISO (ISO LAC) compared to KET (KET LAC; borderline significance; **D**). An increased turnover of DA was detected in mice receiving an intranigral LAC injection under ISO anesthesia compared to sham treatment **(E)**. Sample size is indicated in the graph. Bars represent mean ± SEM. *p < 0.05; ***p < 0.01; ****p < 0.0001 (global lesion effect, two-way ANOVA), ^#^*p* < 0.05, ^##^*p* < 0.01, ^####^*p* < 0.0001 (Tukey’s *post hoc* test vs. corresponding sham-lesioned group). Representative TH photomicrographs of the SNpc in the four experimental groups **(B)**. Rostro-caudal distribution of the number of TH+ cells per section, showing a similar distribution of cell loss after LAC in the ISO and KET groups **(C)**. DA, dopamine; ISO, isoflurane; KET, ketamine; LAC, lactacystin; TH, tyrosine hydroxylase.

LAC infusion into the left SNpc significantly reduced the mean number of dopaminergic cells compared to sham-treatment (*F*_(1,20)_ = 19.88, *p* < 0.001; Figures 1A–C), with no influence of anesthetics on the outcome (*F*_(1,20)_ = 0.002, *p* > 0.05) or interaction effect (*F*_(1, 20)_ = 0.03, *p* > 0.05). These data are supported by the Tukey’s *post hoc* tests as ISO SHAM vs. ISO LAC: *p* < 0.05 and KET SHAM vs. KET LAC: *p* < 0.05. At the level of the striatum, LAC induced a global loss of ipsilateral DA content compared to sham-treatment (*F*_(1,21)_ = 93.95, *p* < 0.0001; Figure 1D) with no anesthetic effect (*F*_(1,21)_ = 0.4371, *p* > 0.05), but with a significant interaction factor (*F*_(1,21)_ = 9.302, *p* < 0.01). *Post hoc* analysis revealed a significant loss of DA in the ipsilateral striatum of KET LAC vs. KET SHAM (*p* < 0.01), as well as ISO LAC vs. ISO SHAM (*p* < 0.0001). In addition, there was a strong trend towards a higher sensitivity (borderline significant) for DA depletion in the ISO LAC group compared to the KET LAC group (ISO LAC vs. KET LAC: *p* = 0.0674; Tukey’s *post hoc* test). As a measure for DA metabolism, we assessed DA turnover in the striatum of mice anesthetized with either ISO or KET. The turnover was calculated as the ratio of both metabolites (DOPAC+HVA) to DA, with a higher ratio indicating a higher DA turnover. Ipsilateral turnover was increased in the LAC-lesioned compared to sham-lesioned mice (*F*_(1,21)_ = 4.344, *p* < 0.05), with no significant anesthetic effect (*F*_(1,21)_ = 3.344, *p* > 0.05), and a strong trend towards an interaction effect (*F*_(1,21)_ = 4.001, *p* = 0.0586). *Post hoc* analysis revealed that this increase was driven by the ISO-treated group (ISO SHAM vs. ISO LAC: *p* < 0.05; Tukey’s *post hoc* test; Figure 1E).

### Motor Function Is Not Influenced by the Choice of Anesthetic

**Figure 2:**
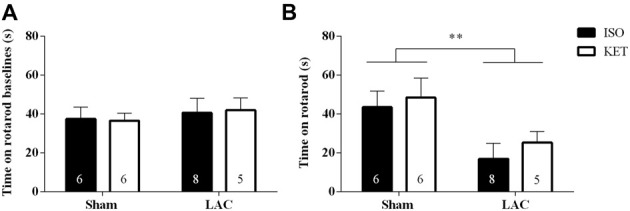
The choice of anesthetic does not influence rotarod performance. No baseline differences could be detected between the treatment groups **(A)**. LAC-injected mice showed a decrease in time spent on the rotarod compared to sham-treated mice, regardless of the anesthetic used **(B)**. Sample size is indicated in the graph. Data are represented as mean ± SEM, **p < 0.01 (global lesion effect, two-way ANOVA). ISO, isoflurane; KET, ketamine; LAC, lactacystin.

No differences were recorded during the rotarod baseline experiments indicating equal acquisition of rotarod motor skills in all groups (Figure 2A). One week following surgery, LAC-injected mice displayed a global impairment in motor coordination and balance compared to sham-treated mice (*F*_(1,21)_ = 8.388, *p* < 0.01), with no significant anesthetic effect (*F*_(1,21)_ = 0.6007, *p* > 0.05), or interaction effect (*F*_(1,21)_ = 0.0359, *p* > 0.05; Figure 2B) present.

## Discussion

Currently, there are no head-to-head comparisons on how anesthetics influence proteasome inhibition-induced neurodegeneration as a model for PD. In this study, we examined for the first time the vulnerability of the nigrostriatal pathway to proteasome inhibition-induced degeneration caused by LAC in mice that were anesthetized with either KET or ISO. Although several studies reported neuroprotective properties of KET *in vitro* and *in vivo* in different disease states (Hudetz and Pagel, 2010; Peltoniemi et al., 2016) as well as in toxin-induced animal models of PD (Datla et al., 2006; Ferro et al., 2007; Fan et al., 2017), we could not observe any difference in LAC-induced neurodegeneration in mice receiving LAC under ISO anesthesia compared to KET. It was shown in rats that anesthesia with KET, compared to thiopental, protected the SNpc against nigrostriatal lesions as well as working memory impairment induced by the neurotoxins 6-OHDA and MPTP (Ferro et al., 2007). In line with these results, Datla et al. (2006) showed a severe loss of dopaminergic cells and striatal DA content caused by injection of 6-OHDA in the left medial forebrain in rats anesthetized with ISO but not with KET (Datla et al., 2006). Although the neuroprotective features of KET are clearly described in several studies, the underlying mechanism is still under debate. Apart from its well-known NMDA blockade, KET disturbs a wide range of intracellular neuronal processes (Sleigh et al., 2014) and inhibits the action of the DA transporter at clinically relevant concentrations, suggesting that this process can enhance monoaminergic transmission (Nishimura and Sato, 1999). In addition, KET may decrease or interfere with the inflammatory cascade as it can suppress lipopolysaccharide-induced cytokine production (Peltoniemi et al., 2016).

Despite the absence of neuroprotective effects of KET at the level of the SNpc in the LAC model, our data show a strong trend towards increased striatal DA loss in mice receiving LAC under ISO compared to KET anesthesia. The effects of anesthetic doses of KET (>100 mg/kg) on DA levels in the striatum of rodents have only been tested in a handful of studies, all reporting negative effects. However, acute KET administration *in vivo* (10–50 mg/kg) is associated with significantly increased striatal DA levels (Kokkinou et al., 2018). The hypothesis of a KET-induced hyperdopaminergic state is supported by Chatterjee et al. (2012), showing increased DA levels and DA turnover in the striatum of mice after acute and chronic treatment. Even more, the significant elevation in DA remained present after a withdrawal period of 10 days after the chronic treatment (Chatterjee et al., 2012).

Besides differences in sensitivity for striatal DA depletion, we demonstrate a significant increase in DA turnover—a parameter used as an index of dopaminergic function (Perez et al., 2008)—in ISO, but not KET-treated mice after LAC. It was shown that anesthetic concentrations of ISO can increase the extracellular concentrations of DA and its metabolites in the striatum of rodents both *in vitro* and *in vivo* (Opacka-Juffry et al., 1991; Irifune et al., 1997; Adachi et al., 2005). However, in these studies, rodents were analyzed several minutes to a maximum of 1 h after exposure and, since our mice were sacrificed 7 days post-lesioning, it seems unlikely that the difference in turnover is still due to a direct effect of the anesthetics. A possible explanation for the increased turnover—thought to reflect a compensatory upregulation of the residual dopaminergic neurons allowing normal function despite significant neurodegeneration (Zigmond et al., 2002; Perez et al., 2008; Blesa et al., 2017)—in the ISO-treated group might be related to the DA loss of these mice. It is known that mice with severe striatal DA loss, which were only present in the ISO-treated group, have higher turnover levels compared to moderate lesioned mice (Blesa et al., 2017). This indicates that only when a certain threshold of DA depletion has been passed, DA turnover becomes significantly elevated and could explain why mice anesthetized with ISO (depletion passed the threshold) have increased turnover, while mice anesthetized with KET have the same turnover as sham-lesioned mice (depletion did not pass the threshold). In line with our results, it was shown that 6-OHDA-lesioned rats showed a compensatory increase in DA turnover, even after a marked decrease in tissue DA levels (Snyder et al., 1990). As an increased DA turnover from spared DA terminals could help to maintain DA homeostasis and help limit the parkinsonian symptoms, this might explain the relative absence of deficits in motor function in our mice with a severe DA depletion in the ISO-treated group compared to moderate lesioned mice in the KET-treated group.

Altogether our data suggest that LAC-induced neuronal death is not dependent on the anesthetic used during surgery. On the contrary, an effect of anesthetic on striatal DA content was present as DA depletion was slightly less pronounced in mice anesthetized with KET compared to ISO. In conclusion, KET does not prevent nigrostriatal degeneration induced by proteasome inhibition. Yet, given the observed effects on DA content and DA turnover, it is still recommended to use the same anesthetic within one experimental set-up.

## Author Contributions

LD, AM and EB designed the experiments; wrote the manuscript. LD performed the experiments. All authors reviewed and commented on the manuscript and approved it in its final form.

## Conflict of Interest Statement

The authors declare that the research was conducted in the absence of any commercial or financial relationships that could be construed as a potential conflict of interest.

## References

[B1] Adachi Y. U. Yamada S. Satomoto M. Higuchi H. Watanabe K. Kazama T. (2005). Isoflurane anesthesia induces biphasic effect on dopamine release in the rat striatum. Brain Res. Bull. 67, 176–181. 10.1016/j.brainresbull.2005.06.020 16144652

[B2] Bentea E. Van der Perren A. Van Liefferinge J. El Arfani A. Albertini G. Demuyser T.. (2015). Nigral proteasome inhibition in mice leads to motor and non-motor deficits and increased expression of Ser129 phosphorylated α-synuclein. Front. Behav. Neurosci. 9:68. 10.3389/fnbeh.2015.00068 25873870 PMC4379937

[B3] Blesa J. Trigo-Damas I. Dileone M. del Rey N. L. G. Hernandez L. F. Obeso J. A. (2017). Compensatory mechanisms in Parkinson’s disease: Circuits adaptations and role in disease modification. Exp. Neurol. 298, 148–161. 10.1016/j.expneurol.2017.10.002 28987461

[B4] Chatterjee M. Verma R. Ganguly S. Palit G. (2012). Neurochemical and molecular characterization of ketamine-induced experimental psychosis model in mice. Neuropharmacology 63, 1161–1171. 10.1016/j.neuropharm.2012.05.041 22683513

[B5] Datla K. P. Zbarsky V. Dexter D. T. (2006). Effects of anaesthetics on the loss of nigrostriatal dopaminergic neurons by 6-hydroxydopamine in rats. J. Neural Transm. 113, 583–591. 10.1007/s00702-005-0353-x 16082506

[B6] Fan J.-C. Song J.-J. Wang Y. Chen Y. Hong D.-X. (2017). Neuron-protective effect of subanesthestic-dosage ketamine on mice of Parkinson’s disease. Asian Pac. J. Trop. Med. 10, 1007–1010. 10.1016/j.apjtm.2017.09.014 29111184

[B7] Ferro M. M. Angelucci M. E. M. Anselmo-Franci J. A. Canteras N. S. Da Cunha C. (2007). Neuroprotective effect of ketamine/xylazine on two rat models of Parkinson’s disease. Braz. J. Med. Biol. Res. 40, 89–96. 10.1590/S0100-879X2006005000053 17225001

[B8] Fu Y. H. Yuan Y. Halliday G. Rusznák Z. Watson C. Paxinos G. (2012). A cytoarchitectonic and chemoarchitectonic analysis of the dopamine cell groups in the substantia nigra, ventral tegmental area and retrorubral field in the mouse. Brain Struct. Func. 217, 591–612. 10.1007/s00429-011-0349-2 21935672

[B9] Hemmings H. C. Jr. Akabas M. H. Goldstein P. A. Trudell J. R. Orser B. A. Harrison N. L. (2005). Emerging molecular mechanisms of general anesthetic action. Trends Pharmacol. Sci. 26, 503–510. 10.1016/j.tips.2005.08.006 16126282

[B10] Hudetz J. A. Pagel P. S. (2010). Neuroprotection by ketamine: A review of the experimental and clinical evidence. J. Cardiothorac. Vasc. Anesth. 24, 131–142. 10.1053/j.jvca.2009.05.008 19640746

[B11] Irifune M. Sato T. Nishikawa T. Masuyama T. Nomoto M. Fukuda T.. (1997). Hyperlocomotion during recovery from isoflurane anesthesia is associated with increased dopamine turnover in the nucleus accumbens and striatum in mice. Anesthesiology 86, 464–475. 10.1097/00000542-199702000-00022 9054265

[B12] Kokkinou M. Ashok A. H. Howes O. D. (2018). The effects of ketamine on dopaminergic function: meta-analysis and review of the implications for neuropsychiatric disorders. Mol. Psychiatry 23, 59–69. 10.1038/mp.2017.190 28972576 PMC5754467

[B13] Mackey S. Jing Y. Flores J. Dinelle K. Doudet D. J. (2013). Direct intranigral administration of an ubiquitin proteasome system inhibitor in rat: Behavior, positron emission tomography, immunohistochemistry. Exp. Neurol. 247, 19–24. 10.1016/j.expneurol.2013.03.021 23557600

[B14] Massie A. Schallier A. Kim S. W. Fernando R. Kobayashi S. Beck H.. (2011). Dopaminergic neurons of system x_c_^−^-deficient mice are highly protected against 6-hydroxydopamine-induced toxicity. FASEB J. 25, 1359–1369. 10.1096/fj.10-177212 21191088

[B15] McNaught K. S. P. Belizaire R. Isacson O. Jenner P. Olanow C. W. (2003). Altered proteasomal function in sporadic Parkinson’s disease. Exp. Neurol. 179, 38–46. 10.1006/exnr.2002.8050 12504866

[B16] McNaught K. S. P. Olanow C. W. Halliwell B. Isacson O. Jenner P. (2001). Failure of the ubiquitin-proteasome system in Parkinson’s disease. Nat. Rev. Neurosci. 2, 589–594. 10.1038/35086067 11484002

[B17] Nishimura M. Sato K. (1999). Ketamine stereoselectively inhibits rat dopamine transporter. Neurosci. Lett. 274, 131–134. 10.1016/s0304-3940(99)00688-6 10553955

[B18] Opacka-Juffry J. Ahier R. G. Cremer J. E. (1991). Nomifensine-Induced increase in extracellular striatal dopamine is enhanced by isoflurane anesthesia. Synapse. 7, 169–171. 10.1002/syn.890070210 2011829

[B19] Peltoniemi M. A. Hagelberg N. M. Olkkola K. T. Saari T. I. (2016). Ketamine: A review of clinical pharmacokinetics and pharmacodynamics in anesthesia and pain therapy. Clin. Pharmacokinet. 55, 1059–1077. 10.1007/s40262-016-0383-6 27028535

[B20] Perez X. A. Parameswaran N. Huang L. Z. O’Leary K. T. Quik M. (2008). Pre-synaptic dopaminergic compensation after moderate nigrostriatal damage in non-human primates. J. Neurochem. 105, 1861–1872. 10.1111/j.1471-4159.2008.05268.x 18248617 PMC3264543

[B21] Poewe W. Seppi K. Tanner C. M. Halliday G. M. Brundin P. Volkmann J.. (2017). Parkinson disease. Nat. Rev. Dis. Primers 3:17013. 10.1038/nrdp.2017.13 28332488

[B22] Savolainen M. H. Albert K. Airavaara M. Myöhänen T. T. (2017). Nigral injection of a proteasomal inhibitor, lactacystin, induces widespread glial cell activation and shows various phenotypes of Parkinson’s disease in young and adult mouse. Exp. Brain Res. 235, 2189–2202. 10.1007/s00221-017-4962-z 28439627

[B23] Sleigh J. Harvey M. Voss L. Denny B. (2014). Ketamine—more mechanisms of action than just NMDA blockade. Trends Anaesth. Crit. Care 4, 76–81. 10.1016/j.tacc.2014.03.002

[B24] Snyder G. L. Keller R. W. Jr. Zigmond M. J. (1990). Dopamine efflux from striatal slices after intracerebral 6-hydroxydopamine: evidence for compensatory hyperactivity of residual terminals. J. Pharmacol. Exp. Ther. 253, 867–876. 2110978

[B25] Zigmond M. J. Hastings T. G. Perez R. G. (2002). Increased dopamine turnover after partial loss of dopaminergic neurons: Compensation or toxicity? Parkinsonism Relat. Disord. 8, 389–393. 10.1016/s1353-8020(02)00019-6 12217625

